# Underutilization of Social Insurance among the Poor: Evidence from the Philippines

**DOI:** 10.1371/journal.pone.0003379

**Published:** 2008-10-13

**Authors:** Stella Quimbo, Jhiedon Florentino, John W. Peabody, Riti Shimkhada, Carlo Panelo, Orville Solon

**Affiliations:** 1 School of Economics, University of the Philippines, Diliman, Quezon City, Philippines; 2 Institute for Global Health, University of California San Francisco, San Francisco, California, United States of America; 3 College of Medicine, University of the Philippines, Manila, Philippines; Canadian Agency for Drugs and Technologies in Health, Canada

## Abstract

**Background:**

Many developing countries promote social health insurance as a means to eliminate unmet health needs. However, this strategy may be ineffective if there are barriers to fully utilizing insurance.

**Methods:**

We analyzed the utilization of social health insurance in 30 hospital districts in the central regions of the Philippines between 2003 and 2007. Data for the study came from the Quality Improvement Demonstration Study (QIDS) and included detailed patient information from exit interviews of children under 5 years of age conducted in seven waves among public hospital districts located in the four central regions of the Philippines. These data were used to estimate and identify predictors of underutilization of insurance benefits - defined as the likelihood of not filing claims despite having legitimate insurance coverage - using logistic regression.

**Results:**

Multivariate analyses using QIDS data from 2004 to 2007 reveal that underutilization averaged about 15% throughout the study period. Underutilization, however, declined over time. Among insured hospitalized children, increasing length of stay in the hospital and mother's education, were associated with less underutilization. Being in a QIDS intervention site was also associated with less underutilization and partially accounts for the downward trend in underutilization over time.

**Discussion:**

The surprisingly high level of insurance underutilization by insured patients in the QIDS sites undermines the potentially positive impact of social health insurance on the health of the marginalized. In the Philippines, where the largest burden of health care spending falls on households, underutilization suggests ineffective distribution of public funds, failing to reach a significant proportion of households which are by and large poor. Interventions that improve benefit awareness may combat the problem of underutilization and should be the focus of further research in this area.

## Introduction

Social health insurance is believed to be a powerful instrument for eliminating unmet health needs [1]–[4]. However, even as countries find the resources needed for universal coverage, this alone may not be enough to ensure access and appropriate care. One of the least understood problems is the lack of utilization of social health insurance among the insured [5]–[8]. Studies have shown that patient perceptions of quality of care, cultural, economic, and geographic factors can affect the utilization of health services in social health insurance programs in developing countries [9], [10].

Underuse of insurance can be a problem even among patients receiving care. Transactions costs of filing claims can outweigh insurance benefits when these are low or when there is inefficient administration of insurance benefits. Other drivers of underutilization might include the insured member's lack of awareness on procedures for reimbursement or perhaps on the very concept of insurance. Finally, when benefits are restricted or support values are low, costs are relatively high and insurance benefits are effectively inadequate.

In this paper, we examine the phenomenon of health insurance underutilization, defined as not filing claims despite having legitimate coverage, in the Philippines. The National Health Insurance Program (NHIP), administered by the Philippines Health Insurance Corporation (PhilHealth), has been providing first peso inpatient coverage for close to four decades. The program originally targeted the formal sector and, in 1995, was expanded through national legislation to meet a new mandate of universal coverage. The majority of the members continues to be those with formal employment. In 1997 PhilHealth introduced a special Indigent Program for poor households, where contributions are supported by government subsidies, co-financed by the national and local governments. In 2000, an outpatient benefit package was also introduced. Indigent households continue to be a focus of PhilHealth initiatives aimed at increasing coverage of the poor. One of these is the Quality Improvement Demonstration Study (QIDS). QIDS pilots two interventions which support PhilHealth's objectives of expanding benefits and improving quality of care. QIDS is a policy study involving two policy interventions supportive of government-led health sector reforms [11]. This paper uses data from the QIDS study to estimate the prevalence of insurance underutilization.

## Methods

### Study setting

In 2003, PhilHealth in partnership with the Department of Health, the University of California San Francisco, and UPecon Foundation undertook the Quality Improvement Demonstration Study (QIDS), a multi-level survey of communities, providers, patients and their families [12]–[14].

Data for this analysis comes from the QIDS patient exit interviews conducted in 30 hospitals located in 11 provinces in the central Philippines. The 30 participating hospitals are located in the Visayan island group and the northern tip of Mindanao. These locations were chosen to maintain geographic isolation, i.e., to prevent spillovers into non-experiment sites that are likely to happen if the experiment were conducted in the Luzon island where most provinces have proximity to the nation's capital. QIDS has three experimental arms. Intervention A, is an expanded PhilHealth benefit package for children under 5 years old. To maximize the effectiveness of the intervention, QIDS undertook additional steps to increase enrollment of indigent households into the PhilHealth program. Intervention B provides additional income to doctors and other staff in hospitals that were found to have met pre-set quality standards. In these facilities, PhilHealth payment per physician visit is increased by 66% percent. Every quarter, bonus payouts are computed for each facility, issued to the hospital chief, and distributed to the hospital. The third is a control arm with no interventions other than the ongoing secular trends and is referred to as Intervention C (for control).

The 30 QIDS hospitals covers about 1 million poor households, with an average income of about 65,200 pesos (1,203 USD), about 50 percent below the national mean.

### Data collection

QIDS exit interview surveys were conducted among pediatric patients (6 months - 5 years old) at the time of discharge. The children were subsequently followed up in their homes to gather more detailed socioeconomic data. The surveys obtain information on the confinement and the patients' socioeconomic characteristics, including insurance status. Data were collected in two rounds before and after the A and B interventions were put in place: a baseline round in 2004–2005 and Round 2 in 2006–2007. To monitor interim progress, patient exits were continued on a quarterly basis in 2005–2006 between the two main survey rounds using an abridged version of the instruments. Table 1 presents the sample size, by survey and by intervention and control sites.

**Table 1:** Sample size of patient exit interviews by intervention and control groups.

Survey Round	Intervention Sites	Control Sites
Baseline	1,977	1,012
2^nd^ Quarter 2005	193	86
3^rd^ Quarter 2005	197	92
4^th^ Quarter 2005	184	102
1^st^ Quarter 2006	198	100
2^nd^ Quarter 2006	198	100
Round 2	2,023	1,018

### Analysis

We used quarterly data from the QIDS patient exit interviews to estimate underutilization by computing the proportion of patients with PhilHealth benefits who did not file an insurance claim.

The following model was then used to estimate underutilization, where U, is the probability of filing insurance claims against PhilHealth conditional on being admitted to a QIDS hospital and being a PhilHealth beneficiary:
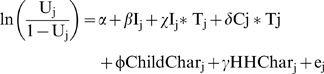
In this model the subscript j refers to the jth patient, I is a dummy variable for the combined intervention sites, C is a dummy variable for control sites, T is a dummy variable for Round 2, ChildCar is a vector of child characteristics (illness, sex, and age), HHChar is a vector of household characteristics (annual household income, parents' education), and e_j_ is the error term that is independently and identically distributed. We used baseline and Round 2 data for this analysis. We used STATA version 10 for all statistical evaluations in this analysis.

## Results

**Figure 1:**
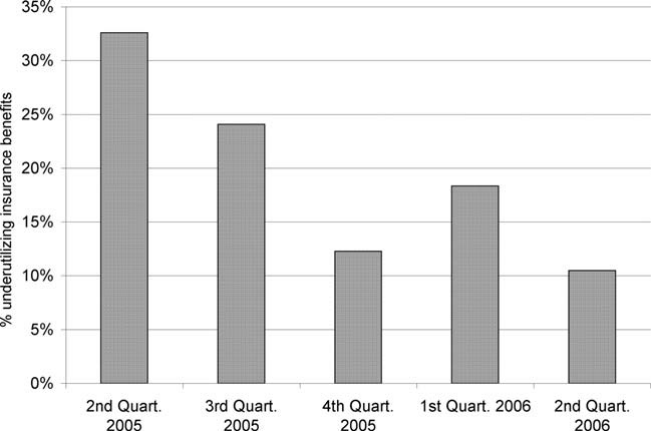
Insurance underutilization in QIDS sites by quarterly monitoring period (2005–2006).

The average underutilization rate obtained for all QIDS study sites across all assessment rounds, was around 15%. The rate was also variable over time. However, we observed a general downward trend over time after the implementation of the interventions which were fully operational by the 3^rd^ quarter of 2005 (Figure 1).

Results from the logistic regression revealed that three factors mitigate against underutilization (Table 2). Parents of discharged children with insurance were less likely to underutilize their insurance (or more likely to file insurance claims for their confinement) if they had a longer length of stay in the hospital or the mother was more educated. When we compared underutilization before and after the study interventions (baseline versus Round 2), we found that in Round 2 patients in the intervention sites were also significantly less likely to underutilize their insurance and more likely to file a claim compared to baseline.

**Table 2:** Logit model of insurance underutilization for children admitted to hospital with PhilHealth insurance.

Variable	Marginal Effects (dPr/dX)	P-value
Intervention	0.03	<0.001
Intervention*Round 2	−0.11	<0.001
Control*Round 2	−0.04	<0.001
Length of stay	−0.02	<0.001
Pneumonia	−0.01	0.40
Diarrhea	0.01	0.63
Age in months	−3.00E-05	0.93
Household income	8.68E-08	0.11
Yrs of father education	1.15E-03	0.51
Yrs of mother education	−0.01	0.01

## Discussion

In this paper, we examined underutilization of the social health insurance program among parents of insured children in the Philippines. QIDS data show that utilization averaged about 85% throughout the study period. After the introduction of the QIDS experimental interventions, which were designed to support PhilHealth's objectives of expanding insurance coverage to poor households and improve quality, we found there was a decline in underutilization. We also found that underutilization is negatively associated with length of stay and as mother's educational attainment. Further, parents of sicker children were more likely to submit claims independent of the two conditions we measured most frequently, diarrhea or pneumonia.

While underutilization has not been widely recognized and may even be surprising amongst the poor, there have been suggestions from previous investigators that this is an important problem. For instance, the underutilization rates we report here have been hinted at in other studies done in the Philippines. In one study, the percentage of members with an approved claim was 5 to 6% for paying members [15]. Another population based survey found that hospital confinement rates among Filipinos ranged between 3–9% depending on the facility type [16]. If we thus assume a 7% hospital utilization rate, to account for the moral hazard of insurance, and compare this to the 5–6% claim probability in the previously referenced study, we would obtain a non-utilization rate ranging from 16–40%.

In the National Demographic and Health Survey (NDHS), a nationwide survey conducted in 2003 by the National Statistics Office, households with PhilHealth coverage and having had experienced illness were asked to list primary reasons for not filing insurance claims. Of the barriers specified, the most often cited ones were not those relating to physical accessibility, such as finding a nearby accredited health facility for services covered by PhilHealth. Rather, the constraints were most often related to those that a member is confronted with during or after confinement, for instance the existence of too many requirements and lack of information on benefits.

In developing countries it is particularly important to understand insurance underutilization because the greatest burden of health care spending falls on households [17]–[20]. In the Philippines, 46.7% of total health care expenditures are accounted for by out-of-pocket payments [21]. Insurance underutilization therefore suggests that there is an ineffective use and distribution of public resources, missing the intended poor households who lose out on financial resources to which they are entitled. Evaluation studies of social insurance program effectiveness around the world typically focus on system problems such as lack of resources or limited coverage rather than the lack of patient information [22]. Underutilization of insurance may be due to the lack of awareness of benefits, potentially high transaction costs relative to potential benefits, or a cumbersome claims process. The political nature of the indigent program in the Philippines can mean household coverage changes year to year; thus households may not be aware of their coverage itself let alone the claims process. This problem may be especially acute for target beneficiaries whose premium payments are fully paid out of public subsidies. Presumably, if insurance premiums are paid by the beneficiaries themselves, there is a natural tendency for the beneficiaries to be educated on the mechanics of the insurance program [23]. In the case of a fully subsidized premium, awareness may be lower. Poor families, therefore, can be expected not to know what preventive and curative services to which they are entitled.

While our study does not provide conclusive information on how to remedy underutilization of insurance, important implications can be drawn. First, targeting less educated mothers and beneficiaries with shorter length of stay is a straightforward place to start in improving utilization. Second, the positive relationship between insurance utilization and QIDS interventions supports the conjecture that increasing awareness of PhilHealth and expanding insurance coverage in either of the two intervention sites increased the likelihood of filing a claim. One common element of the QIDS interventions is information disseminated to various stakeholders about the study and about PhilHealth benefits. Policy Navigators, for example, were deployed in Intervention A sites to increase enrollment; in B sites doctors were touted as being effective and able to provide high quality of care—both of these effects may serve to make patients more aware of the insurance benefits. Social insurance programs may not have sufficient resources to mount effective social marketing campaigns and target special beneficiary groups. In the QIDS interventions sites, the marketing of the intervention themselves may well have extended to the overall reinforcement of PhilHealth insurance benefits. Indeed, other research has shown that if benefits are modest providers do not have the incentive to educate patients about insurance [24]. Another equally important feature of Intervention A is that benefit ceilings were expanded to increase insurance cover to 100%. Thus, even if transaction costs remained high, these are now relatively lower when compared to total insurance benefits with the additional coverage.

We recognize however that an important limitation of this study is that we are unable to identify the causal mechanisms of underutilization. While there are child and household characteristics associated with increased likelihood of insurance claims, there are also uncontrolled confounding factors, especially, in the context of the study design that prevents us from attributing increased utilization to specific aspects of interventions. For instance, in Intervention A sites, benefits expanded to poor households owing to intervention design may also reduce the transaction costs of filing claims in relative terms, making it difficult to disentangle the effects of this with the potential effect due to improved awareness. Furthermore, our study was limited to children admitted as in-patients in public hospitals in the Visayas region, which though is large in size is mostly rural, thus limiting our generalizability to the population as a whole. Further study of underutilization of health insurance using a household survey approach would better elucidate the problem in the general population.

We hope that this surprising finding prompts study of the phenomenon of insurance underutilization in social health insurance schemes around the world. There is also a need to study the effectiveness of information and awareness campaigns to combat this problem. We feel that the key implication to draw from our findings is that a significant amount of resources allocated for improving access to health care is potentially wasted through the underutilization of social insurance. While the exact reasons for this remain unknown, we posit that lack of awareness of is one important obstacle. In the absence of elaborate and well-targeted social marketing campaigns, expanding benefits alone may not be enough to cover the poor.

### Ethics committee approval

Study conducted in accordance with the ethical standards of the applicable national and institutional review boards (IRBs) of the University of the Philippines and the University of California, San Francisco (CHR Approval Number: H10609-19947-05)
